# Effects of Ethnic Settlements and Land Management Status on Species Distribution Patterns: A Case Study of Endangered Musk Deer (*Moschus* spp.) in Northwest Yunnan, China

**DOI:** 10.1371/journal.pone.0155042

**Published:** 2016-05-09

**Authors:** Xueyou Li, William V. Bleisch, Xuelong Jiang

**Affiliations:** 1 State Key Laboratory of Genetic Resources and Evolution, Kunming Institute of Zoology, Chinese Academy of Sciences, Kunming, Yunnan, China; 2 China Exploration and Research Society, Wong Chuk Hang, Hong Kong, PRC; Sichuan University, CHINA

## Abstract

Understanding the status and spatial distribution of endangered species in biologically and ethnologically diverse areas is important to address correlates of cultural and biological diversity. We developed models for endangered musk deer (*Moschus* spp.) abundance indices in and around protected areas inhabited by different ethnic groups in northwest Yunnan China to address different anthropogenic and management-related questions. We found that prediction of relative abundance of musk deer was best accomplished using ethnicity of settlements, conservation status and poaching pressure in an area. Musk deer were around 5 times more abundant in Tibetan regions relative to Lisu regions. We found no significant negative correlates of gathering and transhumance activities on musk deer abundance. Hunting pressure showed no significant differences between protected and non-protected areas, but showed significant differences among ethnic groups. Hunting pressures in areas adjacent to Lisu settlements was 7.1 times more than in areas adjacent to Tibetan settlements. Our findings indicate protected areas in southwest China are not fully effective in deterring human disturbance caused by traditional practices. We suggest that conservation and management strategies should engage traditional culture and practices with a positive conservation impact. Better understanding of indigenous culture may open up new opportunities for species conservation in much wider tracts of unprotected and human-dominated lands. Traditional practices that are not destructive to biodiversity should be allowed as a way of providing a link between the local communities and protected areas thereby creating incentives for conservation.

## Introduction

The effects of traditional culture on biodiversity has received growing attention [1, 2], and it is widely recognized that conservation polices should respect indigenous traditional cultural practices and consider the livelihoods of people affected by conservation restrictions [3–5]. Yet there remains a lack of studies that address correlates of indigenous residents and species abundance in China, and a lack of studies on species abundance across different land management regimes surrounded by different ethnic groups in remote areas.

Ecological factors (e.g. vegetation type, altitude, slope) in combination with human disturbance are often used to predict the distribution of species [6–8]. It is still widely believed that the presence of human communities within sensitive areas is incompatible with viable long-term conservation, and claims of data showing co-existence of human and wild species in the same sites have been controversial [9–11]. Although there is abundant evidence that high levels of some kinds of human activity indeed limit wildlife abundance and species diversity [6, 12], recent empirical research suggests that some forms of traditional livelihood practices may not be adverse to persistence of wild ungulate populations [8, 13].

Northwest Yunnan in southwest China is the area of richest biodiversity in China and may be the most biologically diverse temperate region on earth [14]. The region is a global biodiversity hotspot [15] and encompasses a large UNESCO world heritage natural site. It is also home to diverse indigenous cultures. It retains a high degree of natural character despite thousands of years of human habitation. In northwest Yunnan, native humans and wildlife have coexisted for centuries. Indigenous people have complex traditional livelihood practices, such as nomadic and transhumance agropastoralism among Tibetans, and hunting and gathering among Lisu people. The traditional practices may impose various level of disturbance on wildlife. Human disturbance may affect wildlife survival by altering habitat quality (through collection of firewood, medical plants and timber, livestock grazing), or by direct threat, such as hunting. It has been suggested that a decrease in biological diversity caused by human activities depends on the intensity of disturbance, which can be regulated by proper measures [6].

Musk deer (*Moschus* spp, family Moschidae) are an economically important and highly endangered taxa, distributed throughout the forest and mountainous parts of Asia [16]. The adult musk deer secretes musk, which is several times more expensive than gold, and is widely used in traditional Asian medicine [17]. Musk deer (*Moschus* spp.) have been over-hunted for the use of musk in medicines and perfumes[18], and all seven musk deer species are categorized as Endangered on the IUCN Red List except for *M*. *moschiferus* which is Vulnerable [19].

There are three musk deer species (*M*. *berezovskii*, *M*. *chrysogaster*, and *M*. *fuscus*) in northwest Yunnan [18]. The occupied musk deer habitats in northwest Yunnan are under various kinds of management; some are in protected areas, and some are not. Musk deer populations in variable environmental conditions may exhibit varied responses to habitat disturbance and management. Information on the status and spatial distribution of musk deer populations in biologically and culturally diverse areas is therefore valuable for elucidating correlates of cultural and biological diversity.

We conducted studies of the distribution and relative abundance of musk deer in 3 protected areas and 3 areas without protected area designation in northwest Yunnan using pellet group counts as an index of musk deer abundance. We used a set of predictor variables representing anthropogenic pressures and protected area status to assess the correlates of the musk deer population. Through this study we identified the distribution of musk deer in the region, and identified the main threats that are influencing their abundance. We address the following questions: 1) Are musk deer populations well protected in protected areas? and 2) What predicts the distribution of the species in northwest Yunnan?

## Study Area

The study was carried out in the northern part of Gaoligong Mountain, eastern and western slopes of Biluo Snow Mountain, Longma Mountain, Laojun Mountain and Baima Snow Mountain between 2011 and 2012. The entire area belongs to the Hengduan Mountain Range on the southeast edge of Qinghai-Tibetan Plateau, where parallel ranges of ice-capped mountains—Dandanglika Mountains, Gaoligong Mountains and Kawakarpo/Biluo Snow Mountains—stretch from the north to the south. This unique eco-region constitutes one of the world’s 34 hotspots of biodiversity [20], with a high diversity of ecological niches in a relatively small area [21]. The region also contains a UNESCO World Heritage Site. Southwest China has great cultural diversity, with 25 officially recognized ethnic minority groups comprising 14 million people [3], while Northwest Yunnan is home to over 3 million people belonging to more than 10 ethnic groups [22], Their diverse livelihood practices and traditions may directly impact biodiversity conservation outcomes in a variety of ways. Landscape connectivity and wildlife in the region are negatively affected by illegal hunting and logging, inadequate management planning and lack of clarity of property boundaries [14].

**Fig 1:**
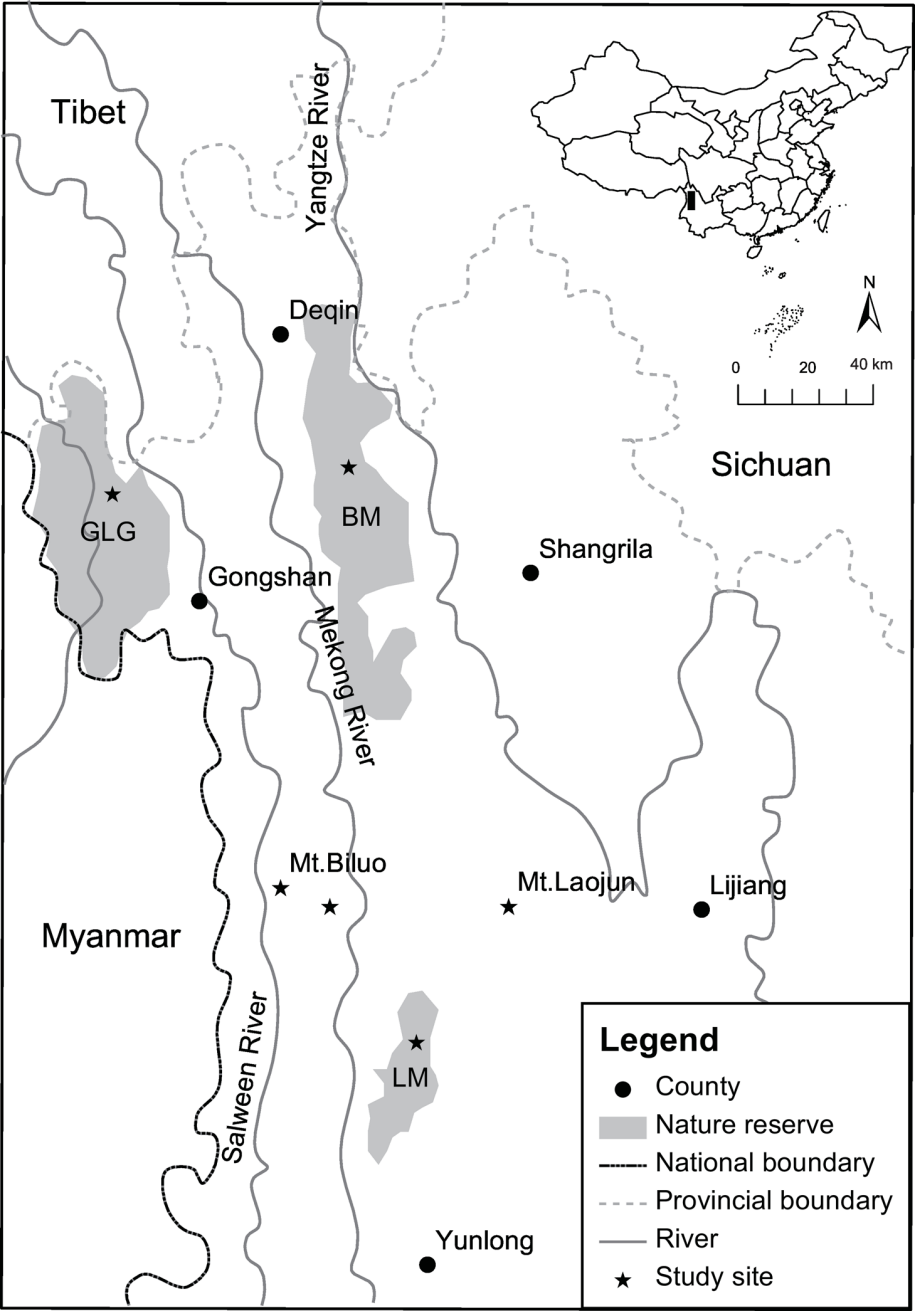
Location of the study sites in northwest Yunnan China. GLG, Gaoligong Mountain Nature Reserve; BM, Baima Snow Mountain Nature Reserve; LM, Longma Mountain of Tianchi Nature Reserve.

We studied the distribution and density of the musk deer population in 3 protected areas and 3 areas without formal protection status. The 6 study areas are isolated from each other and are surrounded by different ethnic groups. Baima Snow Mountain Nature Reserve in Diqing Tibetan Autonomous Prefecture, Gaoligong Mountain Nature Reserve in Nujiang Nu and Lisu Autonomous Prefecture and Longma Mountain of Tianchi Nature Reserve in Dali Bai Autonomous Prefecture are all national level nature reserve. The 3 areas without formal protection were the eastern slope of Mt. Biluo in Diqing Tibetan Autonomous Prefecture, the western slops of Mt. Biluo in Nujiang Nu and Lisu Autonomous Prefecture and Mt. Laojun in Lijiang Naxi Autonomous Prefecture. The six study areas are geographically separated by major river gorges or high mountain ranges (Fig 1).

## Materials and Methods

The study was conducted in the protected areas and adjoining forests of northwestern Yunnan China. The Yunnan Forest Department provided necessary research permits for the study. Since the methods used were non-invasive and relied completely on recording indirect signs of animals, animal care and use committee approval was not required.

As a cryptic, crepuscular species, musk deer are difficult to observe, rendering monitoring programs for conservation purposes a challenge [18]. For elusive ungulate species living in dense forests and/or complex terrain, pellet counts have proved to be a more practical means of estimating population density and abundance [23–26]. Accurate estimates of two parameters, defecation rate and decay rate, are needed to convert fecal pellet group density to population density. These two parameters are influenced by season, climate, habitat type and animal behavior, leading to poor precision and biases in different conditions [25, 26]. Thus, fecal pellet group density, rather than population density, frequently has been used to assess differences among habitats [24, 27–29]. In our study we used fecal group density on transects as an index of relative abundance of musk deer in the region.

### Line transect

We located 55 straight line transects totaling 207.7 km in length within 6 separate forest sites in northwest Yunnan. Transects were designed to cover variation in both forest habitat type and elevation within each sample site. Transect lines were situated in each forest type, each following a compass bearing parallel to slope aspect. Transects were located approximately 2 km apart, which we believe was sufficient for independent sampling across transects [30]. We conducted transect survey only in dry season (February to June in 2011 and 2012) to avoid climatic influences on the dung decay rates.

### Pellet group counting

Musk deer fecal pellets can be accurately discriminated from other sympatric ungulate droppings [30]. Previous study showed that habitats of the three musk deer species in northwest Yunnan overlap [31]. As it impossible to distinguish the three musk deer species from their dung pellets, we pooled the data together to reflect the overall musk deer status in the study areas. The methodology for fecal pellet surveys was adapted from Webbon, Baker [24] and Acevedo, Ferreres [27]. Fecal pellet groups were considered to have decayed totally when six or fewer pellets remained, after which they were excluded from the data. Musk deer relative abundance (*R*) within each transect was calculated as *R* = *N*/*L*, where N is the number of fecal pellet groups recorded per transect, *L* denotes length of transect.

Although fecal pellet counts have been used successfully to estimate ungulate populations [23, 27, 29], results should be interpreted with caution, as they may have been subject to false negatives due to degradation [25]. In our study we conducted all transect counts in the dry, cold season, and the effects of varying rates of fecal decomposition should therefore be limited.

All indications of human activity and presence were noted. On the transects, sightings of people, hunting snares, spent gun cartridge cases, livestock herds, active or abandoned camp sites and signs of medicinal plant gathering were recorded. Human disturbances were classified into 3 categories: gathering, grazing and poaching according to encounters with people or signs observed on transects. Frequencies of disturbances were scored as number of encounters/signs per km walked.

### Statistical analysis

We carried out data analysis in R v. 3.2.1 (R Development Core Team, 2013). We used Independent *t*-tests to examine differences in musk deer pellet group density between protected and non-protected areas and used One Way ANOVA to test for differences among study areas. We used line transects within the 6 study areas as the sampling unit. We used generalized linear models (error distribution family = Gaussian, S1 Fig) to document associations between human disturbance, conservation status of the study areas, main ethnic group living around the study areas and habitat variables, and the abundance of musk deer pellet groups. We examined pair-wise Spearman correlation tests between all variables to check for multicollinearity; if variables were correlated at r_s_≥0.70, only the variables with the lower Akaike’s Information Criterion value were included in further analyses to reduce redundancy [32–34]. We used multimodel inference based on information theory (Akaike’s Information Criterion corrected for small sample sizes, AIC_c_) to assess the relative importance of each predictor. Akaike model weights, *w*_*i*_, were calculated as the weight of evidence in favor of model *i* among the models being compared. The top competing models (within △AIC_c_ = 3.00 of the top model) were included in model averaging (S1 Text).

## Results

The line transect surveys yielded 334 fecal pellet group detections for musk deer. Calculated musk deer relative abundance varied among study areas, ranging from 0.52–4.17 pellet groups/km, with an average of 1.69±1.61 (mean±SD, Table 1).

**Table 1:** Line transects sampled for musk deer relative abundance estimates in 6 areas with different ethnic groups and conservation status in northwest Yunnan China.

Study site*	Location	Ethnic group	Land-use/management rights status	Total transects	Total length of transects (km)	Relative abundance (groups/km)
**GLG**	28°11′N, 98°44′E	Nu	Nature reserve	9	35.1	1.12±0.75
**Eastern slope of BL**	28°22′N, 99°50′E	Tibetan	Community forest	11	47.8	1.71±1.09
**Western slope of BL**	26°33′N, 98°57′E	Lisu	Community forest	8	26.2	0.52±0.35
**LM**	26°23′N, 99°25′E	Lisu	Nature reserve	9	36	0.75±0.53
**LJ**	27°15′N, 99°40′E	Lisu	Community forest	6	23.3	0.53±0.30
**BM**	28°22′N, 99°08′E	Tibetan	Nature reserve	12	39.3	4.17±1.23

* Study sites: GLG = Gaoligong Mountain, BL = Biluo Snow Mountain, LM = Longma Mountain, LJ = Laojun Mountain, BM = Baima Snow Mountain.

**Fig 2:**
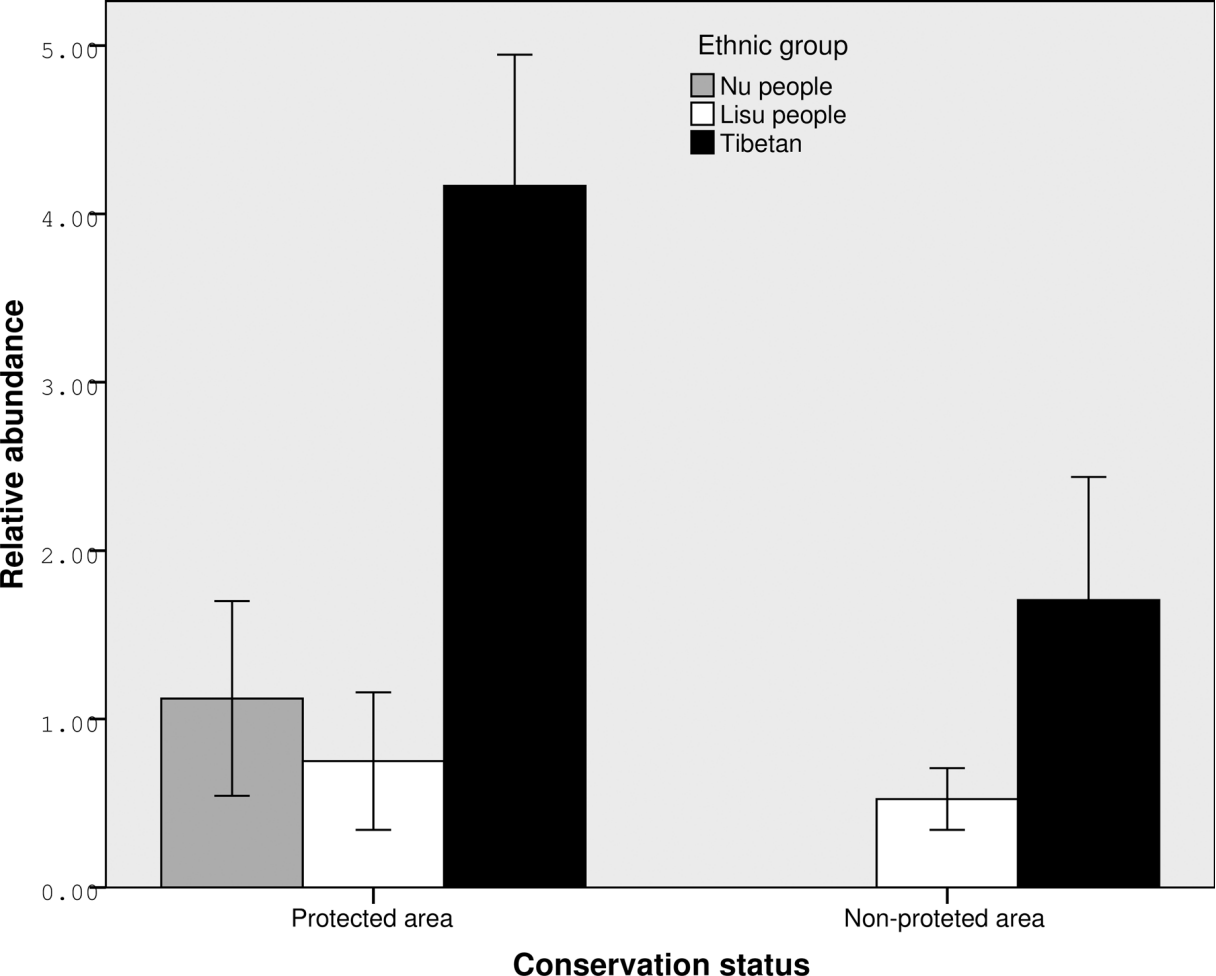
Musk deer relative abundance (Mean±SD pellet groups/km) grouped by ethnic groups and conservation status in northwest Yunnan China.

Musk deer relative abundance showed highly significant differences among study areas (F_5,49_ = 27.82, P<0.001). Between different management classes, the abundance indices in protected areas was significantly higher than that in areas without formal protection (mean±SD, 2.23±1.85 vs. 1.05±0.95 groups per km, t_53_ = 2.89, P = 0.006). Musk deer relative abundance also showed strongly significant differences across protected areas (F_2,27_ = 43.86, P<0.001) and showed nominally significant differences across areas not under formal protection (F_2,22_ = 7.22, P = 0.004). The abundance indices showed strongly significant differences across ethnic regions (F_2,52_ = 24.92, P<0.001). Tibetan regions held the highest musk deer relative abundance (mean±SD = 2.99±1.69 pellet groups/km), follow by Nu regions and Lisu regions (mean±SD = 1.12±0.75 and 0.61±0.42, respectively). The abundance indices were around 5 times higher in Tibetan regions relative to Lisu regions (Fig 2).

Poaching and grazing were both significantly correlated with ethnic group (2-tailed, poaching: r_s_ = 0.51, P<0.001; grazing: r_s_ = 0.54, P<0.001), but both r_s_<0.7; As the two variables represent the main disturbance in the region and might have important influences on musk deer distribution, the two variables were both retained in the generalized linear regression models. For human disturbances, there were no significant differences in gathering (t_53_ = -0.576, P = 0.576), grazing (t_53_ = 0.013, P = 0.990) and poaching (t_53_ = -1.186, P = 0.242) between protected and non-protected areas. There were no significant differences in gathering among ethnic groups (F_2,52_ = 1.088, P = 0.345), however, grazing (F_2,52_ = 11.252, P<0.001) and poaching (F_2,52_ = 40.862, P<0.001) showed highly significant differences among ethnic groups. Areas surrounded by Lisu people held the highest hunting pressures (mean±SD = 0.93±0.43) while areas inhabited by Tibetans had the lowest hunting pressures (mean±SD = 0.13±0.15). As for grazing, Tibetan inhabited areas had the highest occurrence (mean±SD = 1.13±0.57), followed by Lisu and then Nu inhabited areas (Lisu and Nu, mean±SD = 0.59±0.49 and 0.31±0.28, respectively).

A model containing conservation status, native ethnic group and poaching was the most parsimonious model to explain musk deer distribution (indexed by pellet count data), but adding gathering and grazing resulted in competing models (△AICc<3.00, Table 2). These variables were, therefore included in the final averaged model (Table 3, S1 Text).

**Table 2:** Set of linear regression models for musk deer relative abundance, with conservation status (cs), human disturbance (gathering (h1), grazing (h2) and poaching (h3)) and ethnic group of local residents (eth) as explanatory variables.

Model*	K	LL	AIC_c_	△AIC_c_	*w*_*i*_
**cs+eth+h3**	6	-73.43	160.62	0.00	0.42
**cs+eth**	5	-75.66	162.55	1.93	0.16
**cs+eth+h2+h3**	7	-73.24	162.86	2.24	0.14
**cs+eth+h1+h3**	7	-73.24	162.87	2.25	0.14

* The models were ranked by the corrected Akaike information criterion (AIC_c_). K is number of parameters; LL is log-likelihood; △AIC_c_ is difference in AIC_c_ (model score) value; *w*_*i*_ is Akaike model weights. Only models with support (△AIC_c_<3.00) are shown.

**Table 3:** The model-averaged coefficients of the variables predicting the relative abundance of musk deer.*

	Coefficients	SE	z value	P
**Intercept**	1.269	0.402	3.089	0.002
**cs (non-protected area)**	-1.367	0.294	4.542	<0.001
**eth (Lisu people)**	0.736	0.558	1.294	0.196
**eth (Tibetan)**	2.385	0.441	5.280	<0.001
**hd (poaching)**	-0.905	0.438	2.012	0.044
**hd (gathering)**	0.158	0.274	0.564	0.573
**hd (grazing)**	0.132	0.292	0.440	0.660

* The model is averaged across all competing models (△AIC_c_<3.00).

cs, conservation status; eth, ethnic group; hd, human disturbance.

Model-averaging procedures on the remaining descriptors showed that Tibetan ethnic group was the variable showing the highest relative importance. There was a positive and significant effect of Tibetan ethnic group on musk deer relative abundance (coefficient±SE = 2.385±0.441, z = 5.28, P<0.001, Table 3). The conservation status was the second most important variable in predicting musk deer relative abundance. Non-protected status had a negative and significant effect on the abundance indices (coefficient±SE = -1.367±0.294, z = 4.542, P<0.001, Table 3). Considering human disturbances, only poaching had nominally significant effect on the abundance indices (coefficient±SE = -0.905±0.438, z = 2.012, P = 0.044, Table 3). Both gathering and grazing had low averaged-model coefficients, and all P values were above 0.05.

## Discussion

Our results indicate that musk deer abundance in northwest Yunnan varied among study areas, even within protected areas, with a pattern of uneven distribution that suggests that the species is strongly affected by human disturbance. We found musk deer abundance in protected area was significantly higher than in areas without formal protection, indicating that protected areas are important for musk deer populations. Habitat fragmentation as well as poaching are prevalent outside of protected areas and may account for the species’s current distribution. Our findings support the importance of locating protected areas in remote and thereby passively protected sites [35].

It is widely believed that conservation can be successful only in protected areas where human use is legally excluded [36, 37]. Thus, it is not surprising that musk deer abundance is higher in protected areas than in areas without formal protection. However, significant differences in musk deer relative abundance across protected areas indicates that there are other factors affecting the species, not merely conservation status. Model selection showed musk deer distribution is affected by surrounding ethnic groups. The most important variable in predicting musk deer distribution is the ethnic group of adjacent human inhabitants; areas inhabited by Tibetans uniformly showed high musk deer relative abundance. In non-protected areas, musk deer relative abundance was relatively low, except in Tibetan inhabited regions.

Our results reveal for the first time the significant positive correlation of Tibetan ethnic group and musk deer abundance, which has important implications for conservation. This raises important questions for the future–why are some cultures more effective at conservation than others? Consistent with Xu and Melick [3], our results showed that poaching and grazing were both significantly correlated with ethnic group, which implies wildlife species in areas inhabited by the same ethnic group might be under similar kinds of human disturbances. Some traditional cultures are known to have promising potential for enhancing biodiversity conservation [2, 5, 38]. Traditional Tibetan culture has profound impacts on local people’s attitudes and behaviors toward the protection of habitats and wildlife mainly through two traditions: protecting sacred sites and prohibiting hunting [38].

Ethnic groups should be understood as complex entities with differentiated custom and livelihood practices. These in turn influence levels of disturbance to biodiversity. Management strategies should be supportive of traditional cultures that have a positive conservation impact. In future conservation planning and management of this area of Yunnan, it is crucial to recognise and involve ethnic communities to achieve both conservation objectives and community development. Studies on traditional cultural practices in order to elucidate other possible avenues of contributing effectively and efficiently to conservation should be encouraged.

In our study, we recorded both spot sighting and signs/tracks of human activities encountered along transects, providing indices that combine past and present disturbance. Our model averaged results suggest that poaching is a major threat to musk deer populations. This mirrors previous findings about musk deer population depletion [16, 18]. Throughout its distribution range, poachers in northwest Yunnan mainly use snares to trap and kill musk deer, and thus it is difficult for local management offices to completely stop illegal hunting. Our results showed there were no significant differences in frequencies of poaching between protected and non-protected areas. Grazing and collection of non-timber forest products are also prevalent throughout northwest Yunnan, regardless of official restrictions inside nature reserves.

This suggests that protected areas in this region were not effective in deterring these kinds of human disturbances. In contrast, ethnic group is significantly correlated with hunting pressure on wildlife in northwest Yunnan. Hunting pressures in areas where surrounding inhabitants were Lisu people were 7.1 times more than those in areas surrounded by Tibetans.

Under moderate human disturbance, many mammal species can demonstrate behavioral adaptability to living near people, for example, by becoming more secretive and nocturnal [39]. In our study, seasonal gathering and nomadic grazing showed no significant negative correlation with musk deer abundance. Previous studies also found moderate transhumance agricultural activities showed no negative effects on certain wildlife populations [4]. Pastoralists in northwest Yunnan graze their livestock in alpine meadows in summer and autumn and drive them back to villages in winter and spring, sharing space with mammalian fauna. However, the negative impacts of extensive grazing must also be considered. Unmanaged grazing can negatively impact ecosystems and wildlife populations through, for example, overgrazing [40] and deforestation [41].

## Conclusion

Musk deer abundance in northwest Yunnan is affected by surrounding ethnic group conservation status and levels of poaching. Human disturbance level is significantly correlated with ethnic group. Protected areas in southwest China are not fully effective in deterring human disturbance caused by traditional practices. Thus, conservation synergies should be promoted by recognising, involving and enhancing traditional culture and practices that have a positive conservation impact. In contrast, traditional culture and practices with a negative conservation impact should be better understood as a basis for finding solutions. Prohibitive laws should be relaxed to allow sustainable uses (non-timber collection and nomadic grazing) that are not destructive, as a way of providing links between local communities and protected areas and thereby creating incentives for conservation.

We agree with Geldmann, Barnes [11] that protected areas are essential, but their coverage is intrinsically limited. Nature reserves in Yunnan Province only cover about 7% of the total landscape, less than half of the average coverage of China (15%) [42]. Better understanding of indigenous knowledge, values and practices, and their implications for wildlife, may open up new opportunities for species conservation in much wider tracts, including unprotected and human-dominated landscapes.

## Supporting Information

S1 Fig Normal probability plot of the response variable. (TIF)

S1 Text Model selection table and model-averaged coefficients for competing models. (TXT)
