# Supplementary figures and images for: Effects of Ethnic Settlements and Land Management Status on Species Distribution Patterns: A Case Study of Endangered Musk Deer (Moschus spp.) in Northwest Yunnan, China
Source: PLoS One. 2016 May 9;11(5):e0155042. doi: 10.1371/journal.pone.0155042 (PMC4861270; doi:10.1371/journal.pone.0155042)

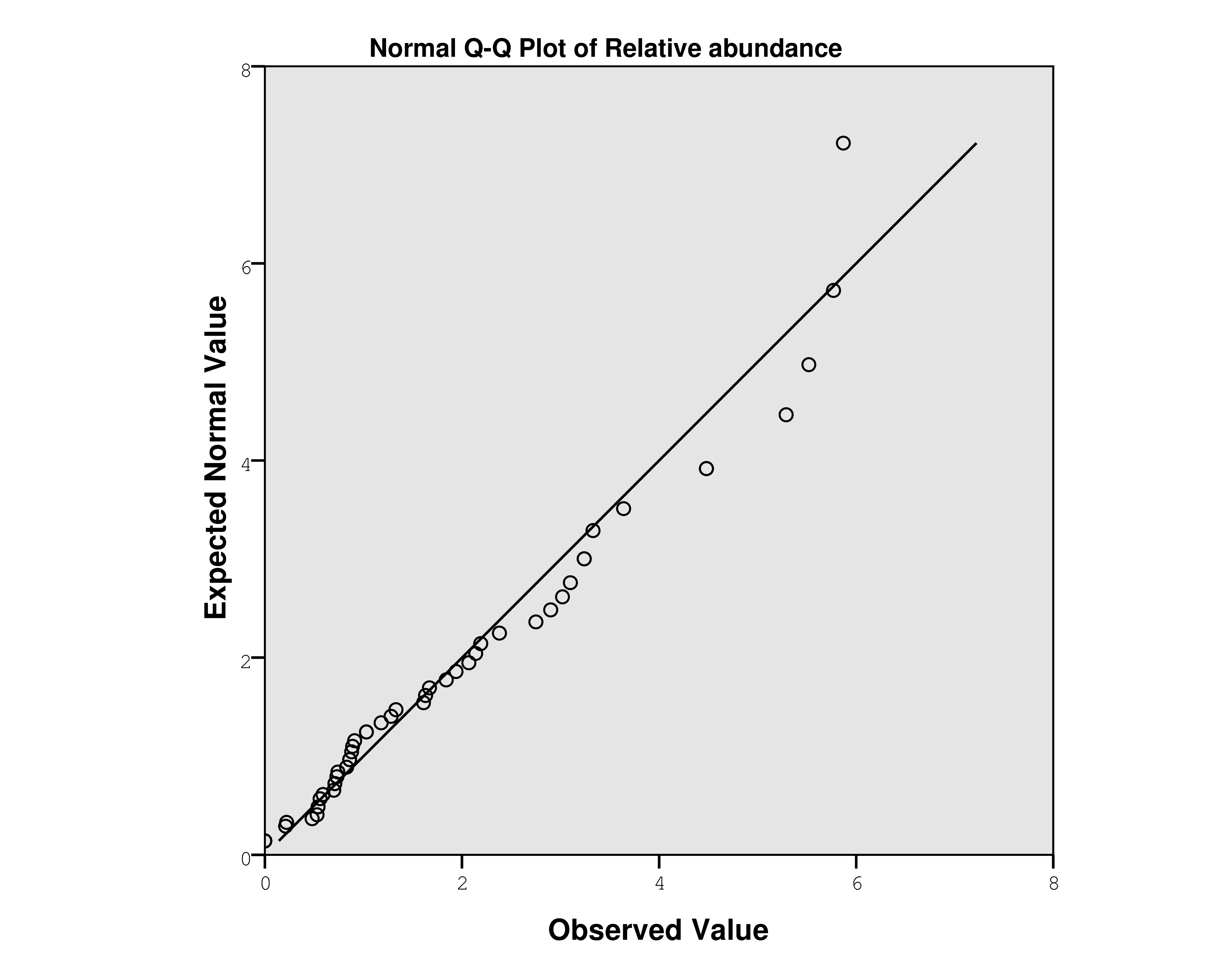

Supplement: S1 Fig — (TIF) [file pone.0155042.s001.tif]
